# Functional plasticity of the *N*-methyl-d-aspartate receptor in differentiating human erythroid precursor cells

**DOI:** 10.1152/ajpcell.00395.2014

**Published:** 2015-03-18

**Authors:** Pascal Hänggi, Vsevolod Telezhkin, Paul J. Kemp, Markus Schmugge, Max Gassmann, Jeroen S. Goede, Oliver Speer, Anna Bogdanova

**Affiliations:** ^1^Institute of Veterinary Physiology, University of Zurich, Zurich, Switzerland;; ^2^Division of Pathophysiology and Repair, School of Biosciences, Cardiff University, Cardiff, United Kingdom;; ^3^Division of Hematology University Hospital Zurich, Zurich, Switzerland;; ^4^University Children’s Hospital, Zurich, Switzerland;; ^5^Children's Research Center, Zurich, Switzerland;; ^6^Zurich Center for Integrative Human Physiology (ZIHP), University of Zurich, Zurich, Switzerland

**Keywords:** calcium, differentiation, erythropoiesis, NMDA receptor

## Abstract

Calcium signaling is essential to support erythroid proliferation and differentiation. Precise control of the intracellular Ca^2+^ levels in erythroid precursor cells (EPCs) is afforded by coordinated expression and function of several cation channels, including the recently identified *N*-methyl-d-aspartate receptor (NMDAR). Here, we characterized the changes in Ca^2+^ uptake and electric currents mediated by the NMDARs occurring during EPC differentiation using flow cytometry and patch clamp. During erythropoietic maturation, subunit composition and properties of the receptor changed; in proerythroblasts and basophilic erythroblasts, fast deactivating currents with high amplitudes were mediated by the GluN2A subunit-dominated receptors, while at the polychromatic and orthochromatic erythroblast stages, the GluN2C subunit was getting more abundant, overriding the expression of GluN2A. At these stages, the currents mediated by the NMDARs carried the features characteristic of the GluN2C-containing receptors, such as prolonged decay time and lower conductance. Kinetics of this switch in NMDAR properties and abundance varied markedly from donor to donor. Despite this variability, NMDARs were essential for survival of EPCs in any subject tested. Our findings indicate that NMDARs have a dual role during erythropoiesis, supporting survival of polychromatic erythroblasts and contributing to the Ca^2+^ homeostasis from the orthochromatic erythroblast stage to circulating red blood cells.

every hour, approximately 10^10^ new erythrocytes are released into the circulation in an adult human after having undergone a tremendous variety of critical transformations during erythropoiesis. These sequential transformations occur within several days and are controlled by numerous intracellular and extracellular regulatory factors. Erythropoietin (Epo) and Ca^2+^ ions are two of the essential players in signaling cascades that support cell survival, proliferation, and differentiation of EPCs through stages from burst-forming units-erythroid (BFUe) to red blood cells (RBCs) (9, 30, 53, 61). The key role of stage-specific alterations in intracellular Ca^2+^ levels and the underlying changes in activity of Ca^2+^ transport pathways in the EPCs have been reported previously (21, 42, 53). Omission of extracellular Ca^2+^ completely inhibits erythopoietic maturation (44). Given its importance, the molecular identity of ion channels mediating Ca^2+^ uptake by EPCs during the early stages of maturation has been a subject of intensive investigation (11, 15, 42). In human and mouse EPCs, Ca^2+^ influx that is mediated by transient receptor potential channels (TRPCs) contributes to the Epo-Epo receptor (EpoR)-mediated signaling (10, 11, 15, 42, 58). Indeed, Epo regulates, in a concentration-dependent manner, Ca^2+^ influx into already hemoglobinized BFUe-derived EPCs, but not in EPCs at earlier stages of differentiation (24). However, Cheung et al. (8) have reported that Ca^2+^ influx induced by Epo was insufficient to activate Ca^2+^-sensitive K^+^ channels (Gardos channel). This strongly indicates the presence of multiple Ca^2+^ uptake pathways in EPCs during differentiation (52). We have recently reported the presence of *N*-methyl-d-aspartate receptors (NMDARs) in human progenitor cells and have shown that these nonselective, ligand-gated cation channels mediate Ca^2+^ uptake in EPCs (18, 35).

NMDARs are best characterized in neurons, but are not limited to the central and peripheral nervous system alone. Indeed, these receptors have been reported in a variety of cells and tissues, including osteoclasts, megakaryocytes, leucocytes, and RBCs (28, 35, 37, 39, 41). NMDARs are formed of glycine-binding subunits GluN1 and GluN3A/3B, and glutamate-binding subunits GluN2A/2B/2C/2D (12, 46, 59). For full activation, the heteromeric ion channel requires glutamate and the cotransmitters glycine (in the brain) or d-serine (in the spinal cord) (12). Depending on the subunit composition, cation selectivity, deactivation time, conductance, sensitivity to Mg^2+^ inhibition, and pharmacology of the NMDARs vary greatly (6, 46, 50). NMDARs containing GluN2C/2D have a lower conductance and a prolonged decay time (45, 47). Combination of these subunits with GluN3A/B results in a further decrease in the current amplitude, distinct channel properties, and altered pharmacology (6, 7, 34). In contrast, NMDAR containing the GluN2A subunit has a fast decay time and higher conductance (55). Recently, we have shown that expression of the *GRIN2C* (GluN2C) and *GRIN3B* (GluN3B) mRNA levels increased progressively from proerythroblast to orthochromatic erythroblast stage, whereas *GRIN2A* gene, coding for the GluN2A subunit, exhibited a high interindividual variability during maturation (35). This study was designed to characterize the function of the NMDARs expressed in EPCs at various stages of differentiation. Using an ex vivo erythropoietic maturation system, we monitored Ca^2+^ uptake and ionic currents induced by NMDAR agonists in human basophilic, polychromatic, and orthochromatic erythroblasts derived from isolated mononuclear cells to determine the stage-dependent alterations in NMDAR channel properties which mirrored the switch in subunit expression pattern observed at the transcriptional level (18, 35). Our new findings demonstrate the importance of the NMDAR in protecting the EPCs against apoptosis.

## MATERIALS AND METHODS

### 

#### Human blood samples and isolation of CD34^+^ and erythropoietic precursor cells.

Blood samples were obtained at the University Children's Hospital Zurich, Switzerland, or mononuclear cells were bought as a product from the Welsh blood bank in Cardiff, UK. All blood donors (*n* = 16 donors, both sexes, ages between 18 and 49 yr, Caucasian) provided written informed consent in accordance with the Declaration of Helsinki. Mononuclear cells were isolated from heparinized venous blood on a Ficoll-Paque PLUS gradient according to the protocol provided by GE-Healthcare (Dietikon, Switzerland).

#### Ex vivo hematopoiesis.

Freshly isolated mononuclear cells were cultured in a two-phase liquid system as described elsewhere (35, 40). During the first phase, cells were maintained in StemSpan Serum-Free Medium for expansion of Hematopoietic Cells (SFEM) containing 0.51 mM l-glutamic acid and 0.4 mM glycine supplemented with StemSpan CC100 Cytokine mixture (StemCell Technologies, Grenoble, France) and 2% of penicillin-streptomycin (Sigma-Aldrich). After 4 days in culture, nonadherent cells were reseeded in StemSpan SFEM containing 20 ng/ml stem cell factor, 5 ng/ml interleukin-3, 1 unit Epo (all provided by ProSpec-Tany Techno-Gene, Ness-Ziona, Israel) and 2% of penicillin-streptomycin (Sigma-Aldrich).

#### Morphological characterization.

Cell morphology was assessed microscopically after cytocentrifugation (Cytospin 4 Cytocentrifuge, Thermo Fisher Scientific, Reinach, Switzerland) and May-Grünwald-Giemsa staining as described elsewhere (40). Differentiation state of the erythropoietic precursor cells (EPCs) was evaluated with the Axio Imager 2 Research Microscope (Carl Zeiss, Feldbach, Switzerland). Standard morphological appearance of basophilic, polychromatic, orthochromatic erythroblasts and reticulocytes is represented in multiple sources (e.g., Ref. 1).

#### Flow cytometry.

To measure the changes in intracellular Ca^2+^ levels, cells were loaded with 3 μM FLUO-4 AM for 30 min, followed by a further 30 min of treatment with the following anti-human monoclonal antibodies: CD34, (eFluor 450 conjugated, clone 4H11, Ref. 48-0349-42), CD71, (APC conjugated, clone OKT9, Ref. 17-0719) both from eBiosciences (San Diego, CA), and CD117 (PC7 conjugated, clone 104D2D1, PN IM3698), CD235a (APC-Alexa Fluor 750 conjugated, clone KC16, PN A89314), and CD45 (Krome Orangeconjugated, clone J.33, PN A96416) all from Beckman Coulter. Loading with both the fluorescent probe and the antibodies was performed in StemSpan SFEM medium (containing 0.51 mM glutamate and 0.4 mM glycine). Preincubation with the receptor antagonist MK-801 (80 μM) for 30 min also occurred in the cell culture medium in a humidified atmosphere with 5% CO_2_ at 37°C. Incubation with FLUO-4 AM, antibodies against the surface markers, and antagonist was performed in cell culture medium to mimic the “physiological conditions” in which basal level of NMDAR activation was maintained. Furthermore, MK-801 can only bind to activated NMDAR. Cell culture medium was replaced by the FACS solution in which cells were washed twice and resuspended before the assessment of fluorescence intensity. FACS solution contained (in mM) 135 NaCl, 5 KCl, 5 HEPES, 10 d-glucose, 2 CaCl_2_ and was adjusted to pH 7.35 with NaOH. Agonist-induced Ca^2+^ uptake was recorded as response to the administration of 150 μM NMDA and 50 μM glycine (NMDA/GLY) to the cell-containing FACS medium. In a separate set of experiments, apoptotic markers were detected in EPCs pretreated with 500 μM MK-801 or memantine for 12 h in glutamic acid- and glycine-containing StemSpan SFEM. Those markers included caspases 3, 8, 9 and phosphatidylserine. Unstained (blank, red histograms) and unstimulated (control, green histograms) cells were used as controls. All experiments have been performed in triplicate, and 15,000 to 35,000 cells had been analyzed at each occasion. Galios Flow Cytometer software was used for data acquisition and Kaluza 1.2 software (Beckman Coulter) was applied for analysis.

#### Electrophysiology.

Electrophysiological experiments were performed using EPCs obtained from eight donors between *day 7* and *20* of erythropoietic maturation. Nonadherent EPCs were plated down on coverglasses coated with poly-l-lysine solution (0.01% vol/wt in H_2_O). Cells were voltage clamped during continuous perfusion at room temperature.

Protocols used elsewhere (14) to record NMDA-induced whole cell currents were adapted for detection of NMDAR activity in EPCs with some modification. The internal solution contained (in mM) 115 *N*-methyl-d-glucamine (NMDG)-HCl, 40 4-(2-hydroxyethyl)-1-piperazineethanesulfonic acid (HEPES), 10 ethylene glycol-bis(β-aminoethyl ether)-*N*,*N*,*N*′,*N*′-tetraacetic acid (EGTA), 2 Na_2_ATP, and 0.2 Na_3_GTP and pH was adjusted to 7.2–7.25 by HCl titration and osmolality to 280–285 mosmol/kgH_2_O. The external solution contained (in mM) 127 NaCl, 20 CsCl, 12 d-glucose, 10 HEPES, 5 BaCl_2_, 2 CaCl_2_, with pH adjusted to 7.35 and osmolality to 300–305 mosmol/kgH_2_O. Cs^+^ and Ba^2+^ were used to reduce K^+^ conductance. High concentration of EGTA was applied to bind the residual free Ca^2+^ after depolarization. To record passive membrane currents, standard intracellular solution containing (in mM) 117 KCl, 11 HEPES, 11 EGTA, 10 NaCl, 2 MgCl_2_, 2 Na_2_ATP, 1 CaCl_2_ adjusted to pH 7.2 with KOH was used. The standard extracellular solution consisted of (in mM) 135 NaCl, 5 KCl, 5 HEPES, 10 d-glucose, 1.5 CaCl_2_, adjusted to pH 7.35 with NaOH. Junction potential was adjusted after seal formation. For single recordings, high-resistance seals (GΩ seals) were formed, and cells were stable for 2–10 min. External and pipette solution (in mM: 117 KCl, 11 HEPES, 10 NaCl, 10 d-glucose, and 1.5 CaCl_2_) was used to record single-channel activity. The pH was adjusted with NaOH to 7.35. Currents were normalized to the cell capacitance (pA/pF). Patch pipettes were pulled from borosilicate glass (GC100F-15, Harvard Apparatus, Holliston, MA) and fire polished. When filled with this pipette solution, they had tip resistances of between 9 and 12 MΩ in the bath solution. Axopatch 200B amplifier, Digidata 1440A, and pClamp 10.3 were used to acquire and filter (5 kHz) data (Axon CNS, Molecular Devices, Downingtown, PA). Leak current in whole cell recordings was subtracted manually. The electrophysiological properties of the EPC membranes were assessed using a voltage-step protocol, which held the voltage at −70 mV and stepped for 200 ms from −120 mV to +60 mV, in 10-mV increments. Agonist-evoked currents were recorded while holding the membrane at −60 mV. A voltage-step protocol described elsewhere (48) with a sequence of a hyperpolarizing step (−100 mV) and two depolarizing steps (+30 mV and +80 mV) was applied to liberate Mg^2+^ from the pore receptor prior to testing of the effects of pore-targeting NMDA antagonists MK-801 and memantine (48). NMDAR modulators were added at the holding potential of −60 mV. The recordings were performed at the depolarizing step (+100 mV). To ensure fast drug application, a Rapid Solution Changer System was used, which allowed solution changes within 20 ms. All chemicals were purchased from Sigma-Aldrich.

#### Statistical analysis.

Data are presented as means ± SD. Statistical analysis was performed using one-way ANOVA with Bonferroni's multiple comparison test, Kruskal-Wallis with Dunn's post test, or Student's paired *t*-test, as appropriate. For all tests, significance was set at *P* < 0.05.

## RESULTS

### 

#### Ex vivo erythropoietic maturation.

**Fig. 1.**
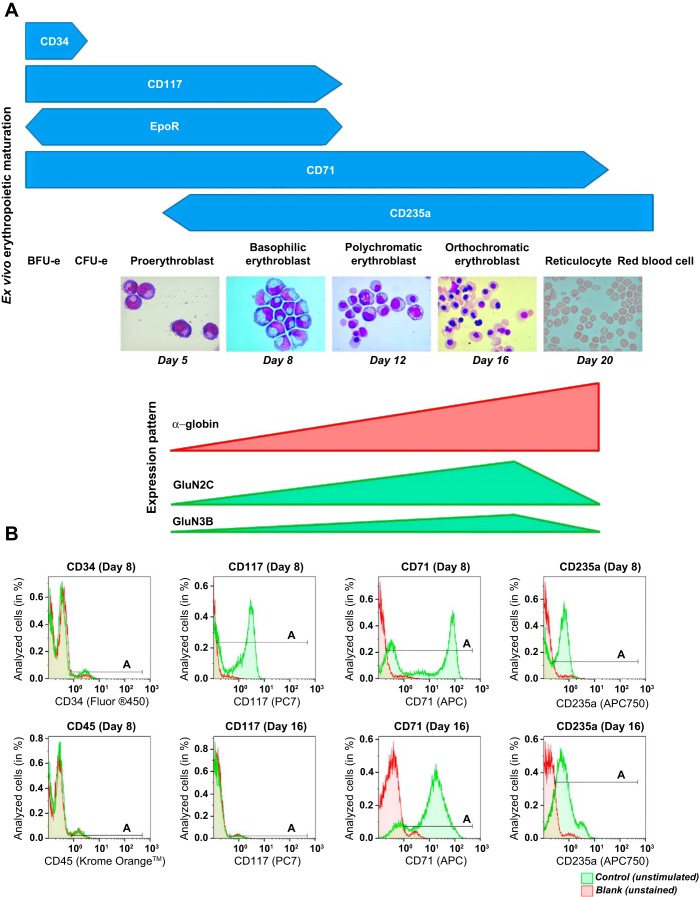
Stage-dependent *N*-methyl-d-aspartate receptor (NMDAR) subunit expression during ex vivo erythropoietic maturation. *A*: differentiation stages of erythroid precursor cells (EPCs) in culture derived from peripheral blood-borne CD34^+^ cells were analyzed morphologically and by the presence of the differentiation markers (CD34, CD117, CD71, and CD235a) assessed by flow cytometry (56) and matched with the α-globin, *GRIN2C*, and *GRIN3B* transcript abundance during ex vivo erythropoietic maturation (36). EpoR, erythropoietin (Epo)-Epo receptor; BFUe, burst-forming units-erythroid; CFUe, colony-forming units- erythroid. *B*: green histograms (control, unstimulated) showing CD34, CD117, CD71, and CD235a abundance in the EPCs at *days 8* (basophilic erythroblasts) and *16* (orthochromatic erythroblasts) in culture. Horizontal bars indicate the A gate selection. Shown in red are the histograms for the unstained cells (blank). CD45 was used to assess the fraction of white blood cells in erythroid cultures (quantification of the data and statistics are presented in Table 1).

Characteristic changes in differentiating EPC cultures were monitored by morphological examination (Fig. 1*A*). In addition, hemoglobin accumulation and the expression of stage-specific markers such as CD34, CD117, CD71, and CD235a were assessed. These differentiation steps were associated with the alteration in the levels of transcripts for the *GRIN* genes (Fig. 1*B*) and corresponding protein abundance of NMDAR subunits (Fig. 1*A*) (18, 35). EPCs are referred to as the dominant (80%) cell type at the specific day as the cell population has never been completely homogeneous. On *day 8* of erythropoietic maturation, 2.3% still expressed CD34, the rest of the EPCs had differentiated to proerythroblasts. White blood cells (CD45^+^) could not be traced. In all experiments, the proportion of CD45^+^ cells was <0.01%. After 8 days in culture, the majority of EPCs had differentiated into basophilic erythroblasts with characteristic morphology (large cells with large nuclei with clumped chromatin, no nucleoli seen) and the onset of CD117^+^ expression. On *day 12*, the majority of the EPCs were smaller in size with further progressing chromatin condensation in the nucleus and low hemoglobinization (Fig. 1*A*). The EPCs expressed CD71 and CD235a and were CD117 negative. These characteristics indicated that at *day 12* the polychromatic erythroblast stage was dominant. On *day 16*, most of the EPCs appeared to be smaller, with highly condensed nuclear and strongly hemoglobinized and expressing CD71 and CD235a and defined as orthochromatic erythroblasts (Fig. 1 and Table 1). Enucleation and reticulocyte formation occurred intensively after *day 17* in culture, with ≥90% of all EPCs losing nucleus by *day 20* as shown in Fig. 1*A*.

**Table 1.** Characterization of erythropoietic maturation with specific surface marker

Day of Maturation	CD34^+^ Cells (Gate A)	CD117^+^ Cells (Gate A)	CD71^+^ Cells (Gate A)	CD235a^+^ Cells (Gate A)	CD45^+^ Cells (Gate A)
Day 8	2.30 ± 1.83%	62.72 ± 14.89%	80.04 ± 9.53%	50.06 ± 11.21%	0.08 ± 0.34%
Day 16	0.00 ± 0.01%	0.77 ± 1.45%	83.12 ± 10.12%	69.69 ± 19.63%	0.00 ± 0.01%

The percentage of cells in gate A (see Fig. 1*B*) presented as mean ± SD (*n* = 9 different donors).

#### NMDA-induced whole cell currents and Ca^2+^ influx in EPCs during differentiation stages.

Activity of NMDARs in basophilic, polychromatic, and orthochromatic erythroblasts was determined by monitoring whole cell currents and Ca^2+^ uptake in a group of healthy human donors that were involved in previous molecular characterization of the receptor (18, 35). Since NMDARs, but not the other ionotropic glutamate receptors, are highly sensitive to *N*-methyl-d-aspartate, we mainly used NMDA for the receptor stimulation. Nonetheless, similar currents were induced by 250 μM glutamate and 50 μM glycine (data not shown).

**Fig. 2.**
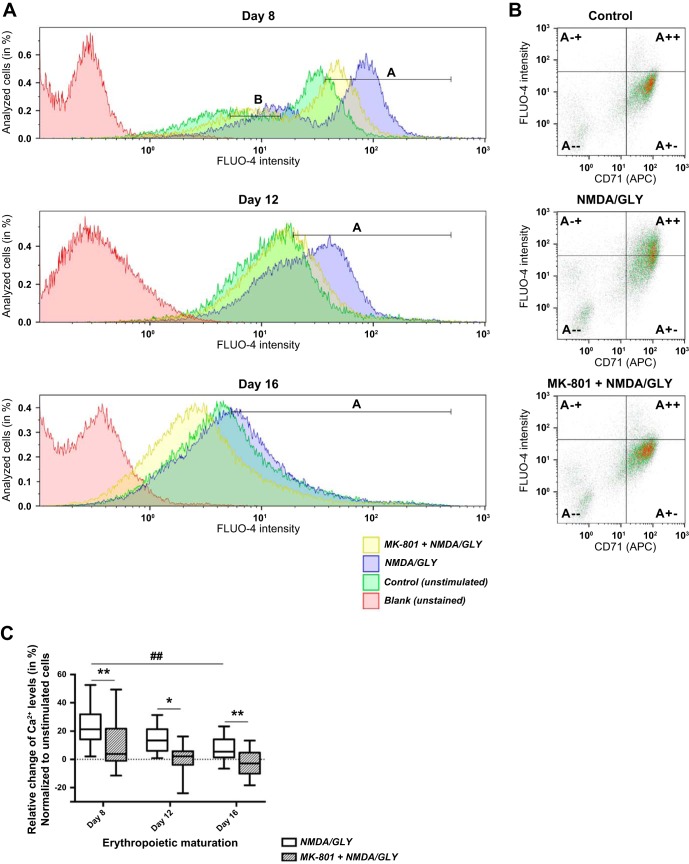
Stage-dependent Ca^2+^ influx into the EPCs upon stimulation with NMDA and glycine (GLY) in the absence or absence of MK-801. The size of cell population responding to the stimulation with NMDA/GLY (150 μM/50 μM) with Ca^2+^ uptake was tested in the EPCs derived from nine different donors at *days 8*, *12*, and *16* in culture. Changes in intracellular Ca^2+^ were evaluated with Ca^2+^ indicator FLUO-4. *A*: representative histograms showing fluorescence intensity in unstimulated cells (control, shown in green), cells stimulated with NMDA (150 μM) and glycine (50 μM) (NMDA/GLY, in blue), and stimulated cells in the presence (MK-801 + NMDA/GLY, in yellow) of antagonist MK-801 (60 μM). Blank (unstained) readouts from the cells free from fluorophore are shown in red. At *day 8* of maturation one population of cells with high FLUO-4 intensity (gate A) and one with low FLUO-4 intensity (gate B) were present. At later stages only one population was present (gate A). Quantification of the data is presented in Table 2. *B*: dot plots illustrating relative change of intracellular Ca^2+^ levels in CD71^+^ cells (gate A*+-* and gate A*++*). Presented are the representative readouts from the EPCs at *day 12* in culture in which Ca^2+^-sensitive fluorescence of FLUO-4 was recorded in unstimulated cells (control), in stimulated (NMDA/GLY) cells, and in stimulated cells in the presence of antagonist (MK-801 + NMDA/GLY). Quantification of the data is presented in Table 3. *C*: Ca^2+^ uptake by the EPCs upon the stimulation with NMDA/GLY on average decreased during maturation from *day 8* to *day 16* in culture (##*P* < 0.01 compared with values for *day 8*). Inhibition of NMDAR by MK-801 resulted in significant reduction of intracellular Ca^2+^ at all stages of maturation (*P < 0.05 and **P < 0.01 compared with the antagonist-free cells). All experiments were performed in triplicate for each of nine donors used in these experiments. The amount of cells analyzed for each single recording ranged from 15,000 to 35,000. Data were normalized to the values obtained for unstimulated EPCs (dashed line). Box plots show the median and 95% confidence interval.

**Fig. 3.**
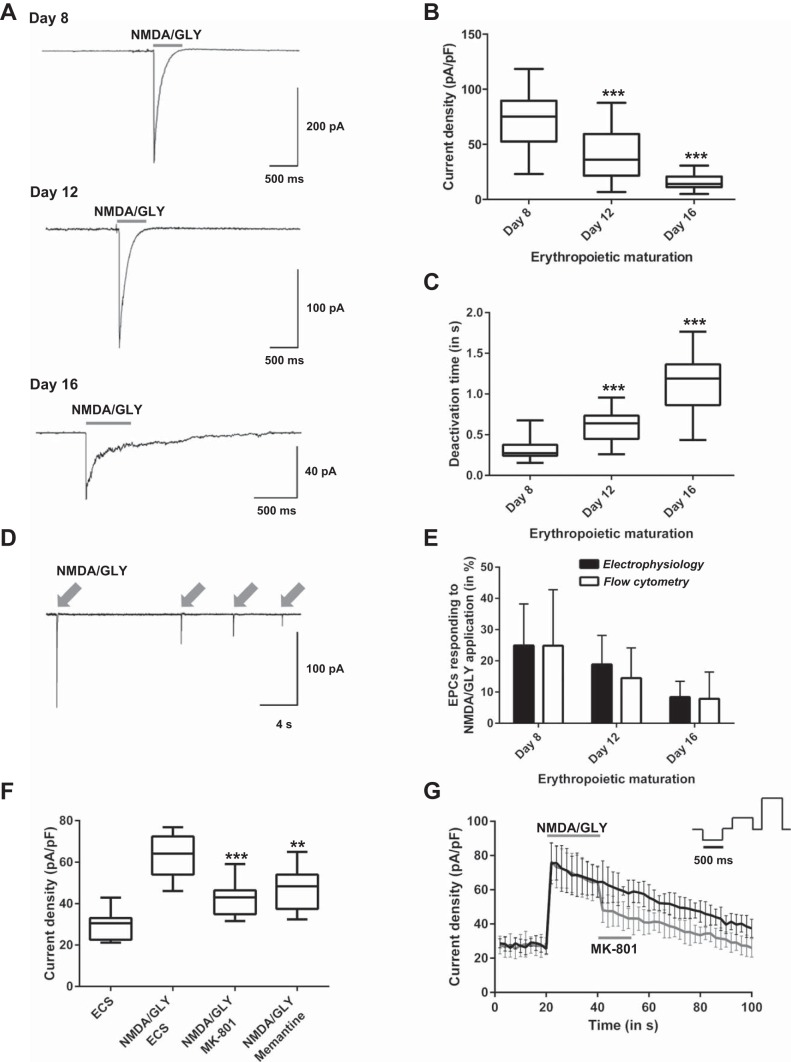
Whole cell currents induced by EPC stimulation with NMDA and glycine. NMDA-induced currents were recorded in whole cell configuration at holding potential −60 mV. *A*: original recordings for the cells exposed to NMDA (150 μM) and GLY (50 μM) at *day 8* (*top*, *n* = 64 cells), *day 12* (*center*, *n* = 59 cells), *day 16* (*bottom*, *n* = 47 cells). *B*: alteration of current density of whole cell currents during erythropoietic maturation (***P < 0.001 compared with *day 8*). *C*: alteration of deactivation time during maturation (***P < 0.001 compared with *day 8*). *D*: deactivation of whole cell currents upon repetitive application of NMDA and GLY in EPCs at *day 12* in culture. *E*: no significant deviation in percentage of EPCs responding to NMDAR activation recorded using flow cytometry and electrophysiology (*P* > 0.05). EPCs from four different donors were analyzed for patch-clamp techniques (*day 8*, *n* = 486 cells; *day 12*, *n* = 458 cells; *day 16*, *n* = 487 cells) and from nine different donors for flow cytometry. *F*: application of the 60 μM MK-801 or 80 μM memantine reduced the current density significantly immediately after induction of NMDA currents (**P < 0.01 and ***P < 0.001). *G*: reduced current density after MK-801 (60 μM) application during repetitive voltage steps were applied. Black trace shows current density without antagonist application after NMDAR activation and gray trace shows current density with MK-801 application after NMDAR activation. All data are means ± SD and were recorded at +100 mV.

Ca^2+^ uptake via the NMDAR was recorded in the cells, which were transferred from the glutamate and glycine-containing culture medium to agonist-free culture medium. Thereafter the cells were stimulated with the agonists (NMDA/GLY). The number of EPCs responding to the stimulation with induction of Ca^2+^ uptake (NMDA/GLY) (exemplified in Fig. 2*A* and Table 2) decreased with differentiation of basophilic erythroblasts to polychromatic and orthochromatic erythroblasts (24.9 ± 17.9%, 14.4 ± 9.6%, and 7.8 ± 8.5% of “responding cells” in the population, respectively) (Fig. 2*C*). All cells responded to stimulation with the NMDAR agonists with an increase in the intracellular Ca^2+^-expressed CD71 (gate A++ in Fig. 2*B* and Table 3). The amplitude of Ca^2+^ uptake in erythroid precursor cells caused by the stimulation with NMDA/GLY reduced with differentiation from basophilic (*day 8*) to orthochromatic (*day 16*) erythroblasts (Fig. 2*C*). In orthochromatic erythroblasts (*day 16*), MK-801 treatment resulted in a modest, but significant decrease in intracellular Ca^2+^ levels (2.5 ± 9.1%) compared with the unstimulated EPCs (dotted line in Fig. 2*C*). Whole cell voltage-clamp recordings were used to verify the data obtained by flow cytometry. At a holding potential of −60 mV, coadministration of NMDA and GLY for 500 ms resulted in induction of currents with characteristics depending on the differentiation stage of the EPCs, as illustrated in Fig. 3*A*. Stimulation of the EPCs in whole cell and single-channel configuration with γ-amino butyric acid (GABA) used as a negative control was without an effect (data not shown).

**Table 2.** Ca^2+^ influx upon stimulation with NMDA/GLY in the absence or presence of MK-801 at various differentiation stages

	Day of Maturation (Gate)							
	Day 8 (Gate A)		Day 8 (Gate B)		Day 12 (Gate A)		Day 16 (Gate A)	
Treatment	FLUO-4 Intensity (Gated Cells)	GMF	FLUO-4 Intensity (Gated Cells)	GMF	FLUO-4 Intensity (Gated Cells)	GMF	FLUO-4 Intensity (Gated Cells)	GMF
Control	18.17 ± 8.19%	11.46	14.66 ± 6.21%	9.25	30.97 ± 7.27%	11.70	59.95 ± 9.73%	4.08
NMDA/GLY	56.06 ± 6.14%	30.83	18.01 ± 2.69%	9.88	60.79 ± 5.88%	21.07	64.71 ± 6.17%	4.46
MK-801 + NMDA/GLY	37.89 ± 7.08%	17.82	16.68 ± 4.82%	9.23	38.94 ± 8.53%	13.39	37.37 ± 7.41%	2.36

Measurements were performed in repetitive erythroid precursor cell EPCs) cultures (*n* = 3) from the same donor. Gating conditions are exemplified in Fig. 2*A* and are presented in the table as the mean percentage of cells in the gate ± SD and geometric mean fluorescence (GMF). NMDA, *N*-methyl-d-aspartate; GLY, glycine.

**Table 3.** Reduction of NMDAR-mediated Ca^2+^ influx after pretreatment with MK-801 in CD71^+^ cells

Treatment	Gate A−− (Gated Cells)	Gate A−+ (Gated Cells)	Gate A+− (Gated Cells)	Gate A++ (Gated Cells)
Control	24.53 ± 4.55%	1.01 ± 0.85%	67.82 ± 7.41%	6.64 ± 5.00%
NMDA/GLY	25.77 ± 3.89%	2.42 ± 1.19%	38.65 ± 4.02%*	33.16 ± 5.03%*
MK-801 + NMDA/GLY	29.02 ± 3.81%	1.63 ± 2.25%	60.72 ± 4.49%	8.63 ± 4.83%

The distribution of cells between the quadrants (see Fig. 2*A*) presented as mean % ± SD.

* *P* < 0.01, compared with the unstimulated control (Student's paired *t*-test).

In basophilic erythroblasts (Fig. 3*A*, *day 8*), stimulation with agonists immediately induced currents with a density of 77.41 ± 31.81 pA/pF (Fig. 3*B*) and a deactivation time of 332.0 ± 149.4 ms (Fig. 3*C*). Desensitization of the channels was observed upon repetitive stimulation with the agonists (Fig. 3*D*). Maturation to polychromatic erythroblasts (Fig. 3*A*, *day 12*) was associated with a decrease in current density to 40.96 ± 25.13 pA/pF (Fig. 3*B*) and prolongation of deactivation to 591.3 ± 206.2 ms (Fig. 3*C*). This tendency persisted with differentiation to orthochromatic erythroblasts (Fig. 3*A*, *day 16*) in which current density declined further to 16.01 ± 7.81 pA/pF (Fig. 3*B*) while deactivation time increased to 1,139.1 ± 382.9 ms (Fig. 3*C*). The number of cells, which were sensitive to agonist stimulation, declined during maturation, from 24.9 ± 13.3% in basophilic erythroblasts to 18.6 ± 9.3% of polychromatic erythroblasts, making up only 8.4 ± 8.6% in orthochromatic erythroblasts. The number of cells responding to the stimulation of NMDARs at various differentiation stages was identical no matter what technique (electrophysiology or flow cytometry) was used for detection of the NMDAR activation (Fig. 3*E*).

#### Sensitivity of agonist-induced currents to MK-801 and memantine.

We have explored the sensitivity of currents induced by the receptor agonists to the potent and selective NMDAR channel blockers MK-801 (60 μM) and memantine (80 μM). As shown in Fig. 3*F*, these noncompetitive antagonists reduced the agonist-sensitive current density significantly. The application of MK-801 decreased the current density mediated by the NMDARs from 62.77 ± 10.42 pA/pF to 42.12 ± 8.51 pA/pF and memantine suppressed the mean current density to 46.82 ± 10.29 pA/pF. Long-term application of NMDA and glycine and repetitive performance of the voltage-step protocol induced deactivation (Fig. 3, *D* and *G*). Further inhibition of current density could be mediated by MK-801 supplementation (Fig. 3*G*).

#### Activation of single channels after application of NMDA and glycine.

**Fig. 4.**
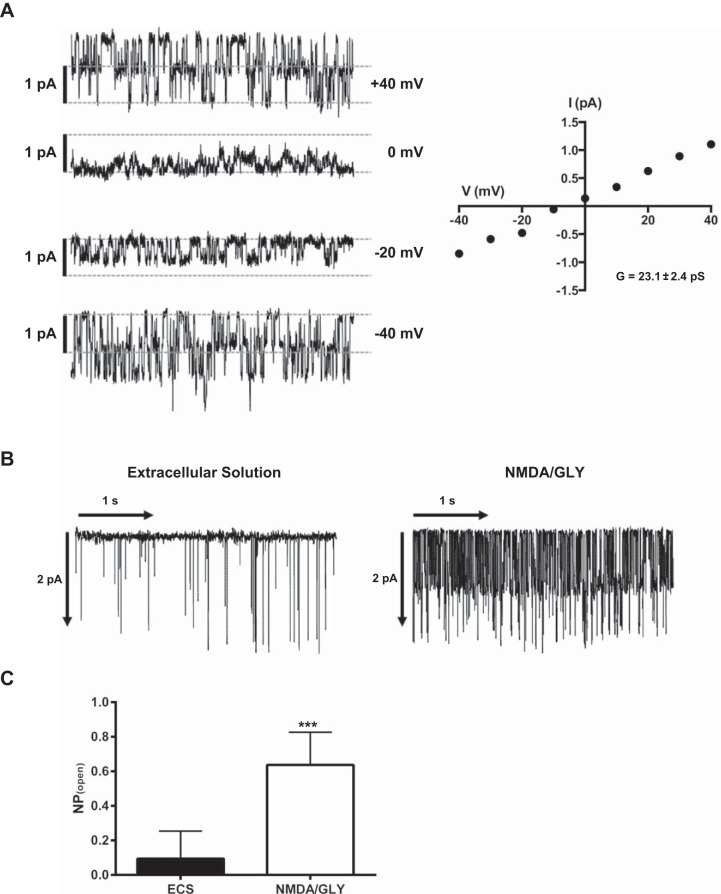
Activation and deactivation of single channels after NMDAR activation. Single-channel recordings were performed in cell-attached configuration to monitor spontaneous single-channel activity and its alteration in response to the stimulation with 150 μM NMDA and 50 μM glycine. *A*: mean single-channel conductance (*G*) was 23.1 ± 2.4 pS (*n* = 4). *V*, voltage; *I*, current. *B*: single-channel recording before (*left*) and after (*right*) NMDA/GLY application. Holding potential was fixed at −40 mV. *C*: change in open probability and number of open channels (*NP*_open_) upon NMDA/GLY application (*n* = 96 cells). ECS, extracellular solution. Data are means ± SD (***P < 0.001).

The effects of NMDAR activation and the concomitant Ca^2+^ uptake on the activity of ion channels is consistent with the properties of Ca^2+^-activated K^+^ channels and were determined in the cell-attached configuration. The single Ca^2+^-sensitive K^+^ channel currents (Fig. 4*A*) had a current-voltage (*I*–*V*) relationship resembling the characteristics of Gardos channels (13). The conductance of 23 pS of the channels was determined from the slope of the regression line. This value was similar to the conductance of 18–22 pS reported for the Gardos channels in mature red blood cells (16, 17, 27). Stimulation of reticulocytes with the NMDAR agonists induced a 6.7-fold increase in the product of open state probability (*P*_open_) and number (*N*) of open channels (*NP*_open_) (0.64 ± 0.19 vs. 0.09 ± 0.16 in nonstimulated cells) in 8.6 ± 7.4% of cells between *day 17* and *day 20* in culture (Fig. 4, *B* and *C*). The single-channel activity decreased after ∼30 s of stimulation with agonists. Removal of the agonists resulted in recovery of the basal channel activity within 2 min. The spontaneous activity of these channels has been previously described by Dyrda et al. (13), and attributed to pressure activation. Further characterization of the Ca^2+^-sensitive K^+^ current was outside the scope of the study.

#### Interindividual variability of NMDAR activity correlates with the changes in GluN subunit expression levels during maturation.

**Fig. 5.**
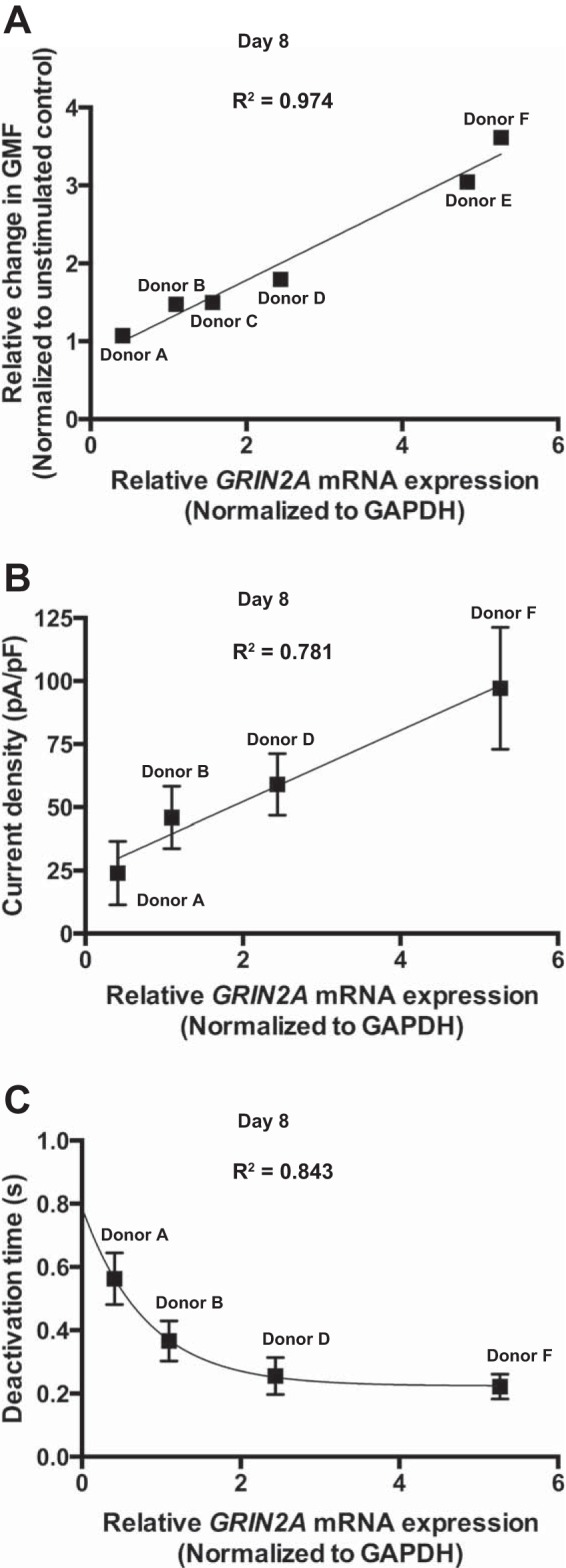
Donor dependence of Ca^2+^ influx, receptor properties, and *GRIN2A* expression during hematopoiesis. mRNA expression was normalized to GAPDH. *A*: correlation between *GRIN2A* mRNA expression and relative change in geometric mean fluorescence (GMF) in the stimulated EPCs. Correlation coefficient was calculated by the change in GMF triggered by the agonists and the relative mRNA expression from six donors. *B*: correlation of current density and relative *GRIN2A* mRNA expression (*n* = 4). *C*: correlation of deactivation time and relative *GRIN2A* mRNA expression from four donors. Data are means ± SD; linear (*A* and *B*) and exponential (*C*) functions were used for curve fitting.

We have monitored interindividual variability in the relative mRNA expression of *GRIN2A* at *day 8* and the relative change of intracellular Ca^2+^ concentration after NMDAR activation that was recorded as a shift in geometric mean fluorescence (GMF). *GRIN2A* expression of individual donors correlated with the amplitude of changes in the intracellular Ca^2+^ levels after NMDAR stimulation (Fig. 5*A*).

**Fig. 6.**
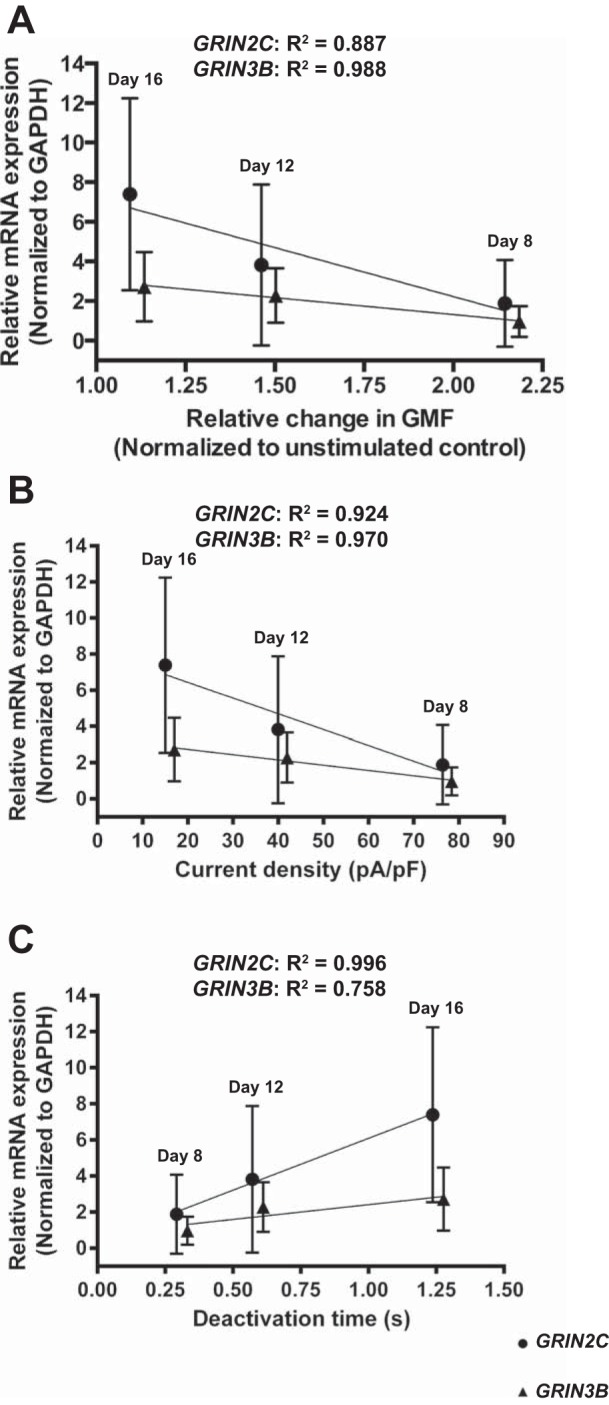
Stage dependence of Ca^2+^ influx, receptor properties, and *GRIN2C*/*3B* expression during hematopoiesis. mRNA expression was normalized to GAPDH. *A*: correlation of relative *GRIN2C* and *GRIN3B* mRNA expression (*n* = 9) and relative change in GMF after NMDAR activation (*n* = 9). *B*: correlation of current density and relative *GRIN2C/3B* mRNA expression during erythropoietic maturation (*n* = 6). *C*: correlation of deactivation time and relative *GRIN2C/3B* mRNA expression during erythropoietic maturation (*n* = 6). Data are means ± SD (correlation coefficients see Table 4).

In basophilic erythroblasts, low levels of the *GRIN2C* and *GRIN3B* transcripts were associated with a prominent increase in the intracellular Ca^2+^ upon NMDAR activation (Fig. 6*A*). Inverse correlation was observed between the upregulation of the *GRIN2C* and *GRIN3B* genes and the amplitude of Ca^2+^ uptake in the EPCs stimulated with the NMDAR agonists during the transformation into polychromatic and orthochromatic erythroblasts (Fig. 6*B*).

Further correlations were detected between the type of NMDA-induced current and the subunit expression pattern. Deactivation time correlated inversely with the relative *GRIN2A* expression levels of individual donors (Fig. 5*C*).

A switch in NMDAR properties from channels with high amplitude and fast decay currents, characteristic of the basophilic erythroblasts, to currents of smaller amplitude and longer deactivation time from polychromatic erythroblast stage onwards, mirrored a switch from the GluN2A to GluN2C/3B in all nine donors (Fig. 6, *B* and *C*).

#### Alteration in electrophysiological properties during enucleation of erythropoietic cells.

Besides the above-mentioned stage-dependent switch in NMDAR properties and the basal intracellular Ca^2+^ levels, alterations in basal electrophysiological properties of the EPC membranes exhibited during enucleation.

**Fig. 7.**
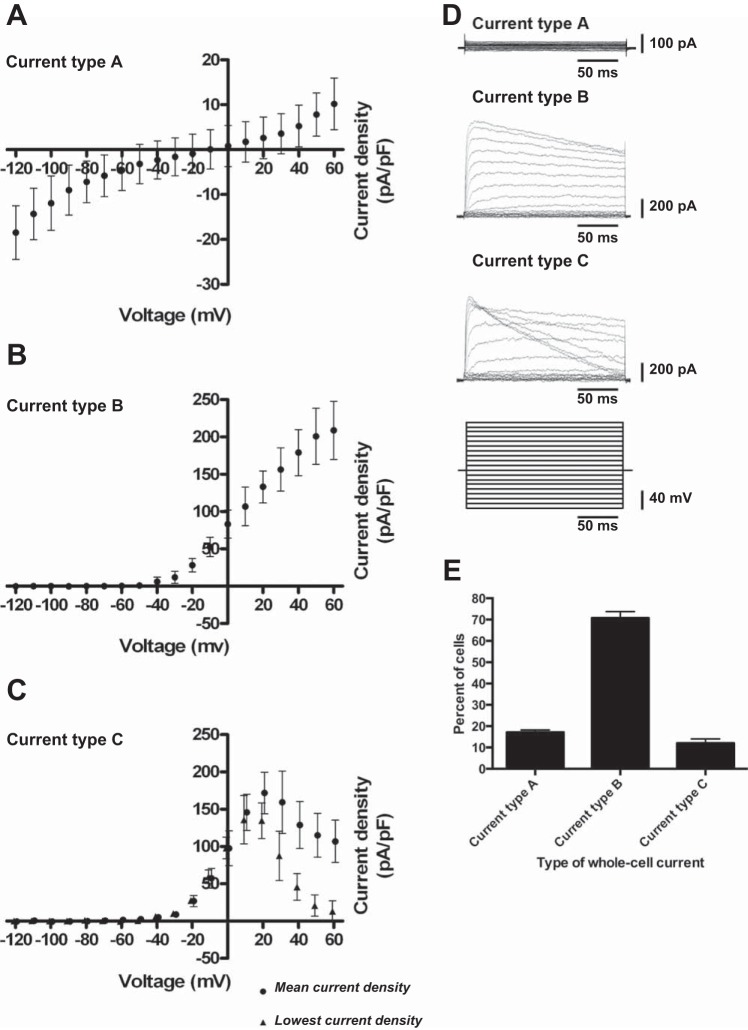
Alteration in electrophysiological properties during hematopoiesis. Cells were voltage clamped in whole cell configuration during hematopoiesis, and voltage steps (10-mV increments) from −120 mV to +60 mV were applied for 200 ms. Current was normalized to cell capacitance (pA/pF) *A*: frequently observed whole cell currents (type A current, 86 cells) from early and intermediate stages of hematopoiesis (until *day 15*). *B*: frequently observed whole cell currents (type B current, 79 cells) from late stage of erythropoietic maturation (from *day 16* on). *C*: infrequently observed type of whole cell currents (type C current, 8 cells) from late stage (*day 18*). *D*: voltage-step protocol and three examples for the three whole cell current types. *E*: analysis of voltage-current relationship from four different donors at *day 18* of differentiation. Data are means ± SD (*n* = 76 cells).

Current-voltage relationship within the −120 to +60 mV potential range showed no substantial changes until *day 15* of differentiation. A representative *I*–*V* curve (current type A) recorded from basophilic and polychromatic erythroblasts showed characteristic lack of voltage dependence within the range of potentials from −90 mV to +30 mV resembling the *I*–*V* relationship reported for chloride channels reported to be present in RBCs (26, 57) (Fig. 7, *A* and *D*). During enucleation (between *day 16* and *18*), a switch from voltage-independent to two types of voltage-dependent behavior occurred in 82.8 ± 1.01% of cells (Fig. 7, *B*–*E*). The majority of cells (70.8 ± 3.01%) exhibited a steep increase in conductance during depolarization from −20 mV to +50 mV (current type B), a feature characteristic of certain potassium channel types (25, 26, 57) (Fig. 7, *B* and *D*). In 12.0 ± 2.01% of EPCs a bell-shaped *I*–*V* relation (type C current) with maximal current density monitored at +30 mV was observed (Fig. 7, *C* and *D*). At *day 18* of differentiation, in 17.2 ± 1.0% of cells the *I*–*V* relationship (type A current) remained essentially voltage insensitive (Fig. 5*E*). Further in-depth studies are required for detailed characterization and molecular identification of all ion channels contributing to these basal electrophysiological properties.

#### Treatment of the EPCs with high doses of NMDAR channel pore blockers induces apoptosis.

**Fig. 8.**
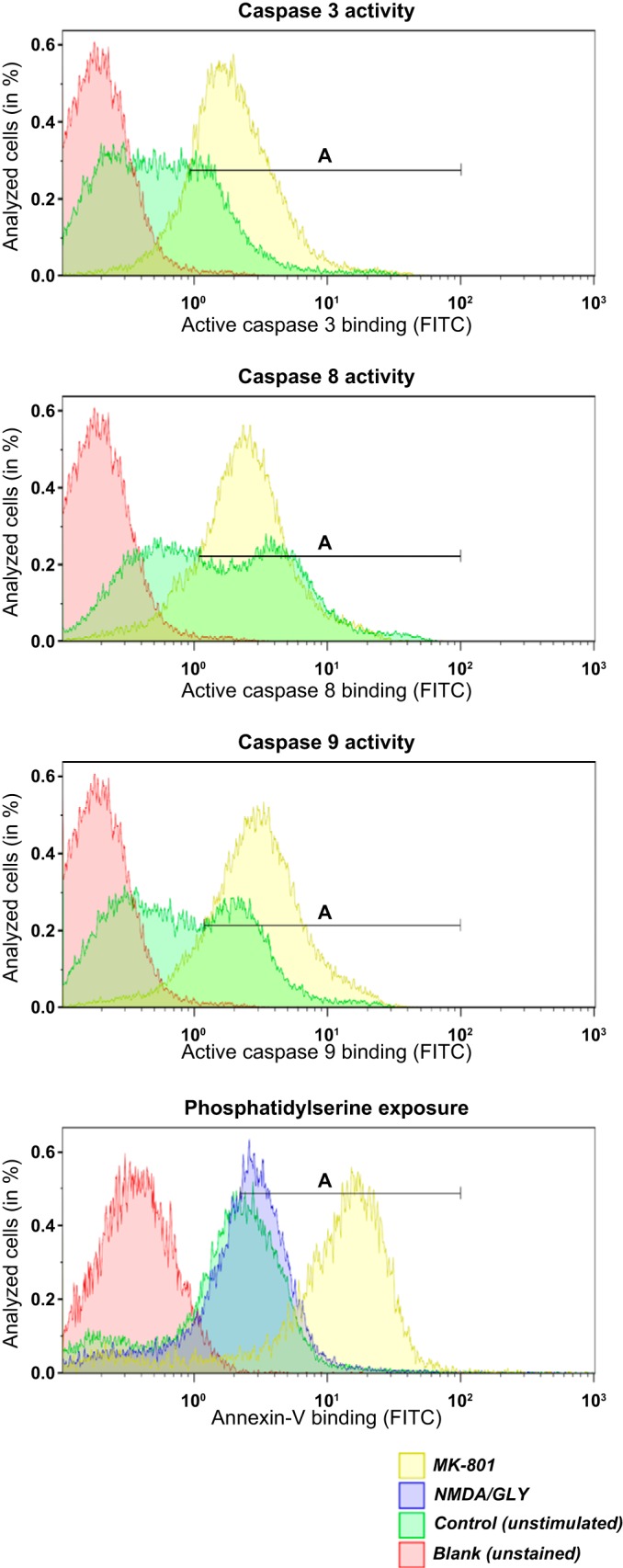
Downstream activation of caspases and enhanced phosphatidylserine (PS) exposure after long-term blockage of NMDAR in differentiating EPCs. EPCs obtained from three donors and cultured for 12 days were incubated for 12 h at 37°C in the presence of 500 μM MK-801 or memantine or were supplemented with NMDA in addition to the medium-borne agonists (510 μM glutamate and 400 μM glycine). Cells were stained with caspase activity marker or FITC-labeled annexin-V antibody. Caspases 3, 8, and 9 were all upregulated (gate A) after long-term incubation (shown in yellow) with MK-801 and memantine (only MK-801 shown). High concentration of NMDA did not affect caspase activity (data not shown). PS exposure was increased (gate A) after long-term inhibition of NMDAR (only memantine shown). Overactivation of NMDAR (NMDA/GLY) with 500 μM NMDA and 100 μM glycine did not influence annexin-V binding (blue histogram) significantly.

Earlier on, we have shown that high doses of memantine or MK-801 (above 100 or 50 μM, respectively) resulted in cell death, which was particularly pronounced for the early differentiation stages (18, 35). Herein we have extended this observation by analyzing the mechanism of cell death induced by high doses of pore-targeting NMDAR blockers. Incubation of basophilic erythroblasts with 500 μM MK-801 or memantine (the dose toxic for both blockers) for 12 h in the SFEM culture medium induced activation of caspase 3, caspase 8, and caspase 9 in the majority of cells (Fig. 8 and Table 4). Phosphatidylserine exposure was enhanced in EPCs exposed to both NMDAR antagonists (Fig. 8 and Table 5). Hyperactivation of the receptors by additional supplementation of NMDA (500 μM) and glycine (100 μM) to the culture medium caused only modest adverse effects (Table 5).

**Table 4.** Induction of caspase activity caused by the treatment of the EPCs with 500 μM of MK-801 or memantine

	Apoptosis Marker					
	Caspase 3 Activity		Caspase 8 Activity		Caspase 9 Activity	
	Gated Cells		Gated Cells		Gated Cells	
Treatment	(Gate A)	GMF	(Gate A)	GMF	(Gate A)	GMF
Control	31.05 ± 7.32%	0.33	49.05 ± 2.60%	0.91	38.63 ± 7.98%	0.47
NMDA/GLY	36.56 ± 10.02%	0.56	53.01 ± 8.92%	1.09	39.91 ± 5.19%	0.53
MK-801	83.95 ± 6.62% †	1.76	79.04 ± 9.15%*	1.94	86.01 ± 8.36% †	2.32
Memantine	74.72 ± 9.19% †	1.59	73.41 ± 8.12%*	1.89	81.56 ± 7.45% †	2.18

The percentage of cells within gate A (Fig. 8) shown as mean ± SD and mean GMF for 3 independent donors.

* *P* < 0.01,

† *P* < 0.001, compared with nonstimulated control (one-way ANOVA).

**Table 5.** Treatment of the EPCs with supra-pharmacological doses of NMDAR channel pore blockers induced Phosphatidylserine (PS) exposure

	Apoptosis Marker	
	Phosphatidylserine Exposure	
	Gated Cells	
Treatment	(Gate A)	GMF
Control	44.3 ± 6.88%	0.82
NMDA/GLY	57.4 ± 5.82%	1.42
MK-801	86.3 ± 14.71%*	5.16
Memantine	84.80 ± 15.16%*	5.02

Percentage of cells in gate A (Fig. 8) presented as mean ± SD and mean GMF for 3 donors.

* *P* < 0.01 compared with the nonstimulated control (one-way ANOVA).

## DISCUSSION

### 

#### Plasticity of NMDAR and whole cell currents during erythropoiesis.

The functional plasticity of the NMDAR during EPC differentiation is the most striking finding of this study. A dynamic switch in the subunit expression patterns was precisely mirrored by changes in the NMDAR channel function. These events contributed to the general remodeling of the membrane reflected by the changes in shape of the *I*–*V* curves observed in the course of EPC differentiation.

During differentiation from basophilic to polychromatic erythroblasts, the GluN2A subunit of the NMDAR in the cells was substituted by the GluN2C subunit, whereas the expression and abundance of the other subunits, of which GluN2D and 3B subunits were dominating, remained unchanged. The electrophysiological properties of NMDAR in EPCs at any of the stages tested exhibited high similarity to those in their counterparts in neurons (49). Namely, proerythroblasts and basophilic erythroblasts exhibited an NMDAR subtype with high amplitude and fast deactivation kinetics reported for the GluN2A-containing receptors (49). These receptors enable efficient glutamate-induced changes in transmembrane potential and massive short-term Ca^2+^ uptake. At these stages of maturation, EPCs were reported to be particularly dependent on Ca^2+^-driven signaling. The latter is activated upon binding of erythropoietin to its receptors (42, 43) and engaged in control of a number of other processes of which iron processing in erythroblasts is one of the most important (20). The peak of Epo receptor expression is associated with the highest NMDA-evoked Ca^2+^ influx, fast deactivation time, and high conductance (Fig. 3, *A–C*) (11, 15, 42). Our findings indicate that prolonged exposure to high antagonist concentration enhances phosphatidylserine exposure (Fig. 8). This supports the hypothesis that the maintenance of NMDAR function is crucial for protection against apoptosis in proerythroblasts and basophilic erythroblasts. These observations are in agreement with the earlier studies in which requirement of the extracellular Ca^2+^ for survival and further differentiation of the EPC was postulated (42). Detailed investigation of the molecular mechanisms of involvement of NMDAR in protection of the EPCs from apoptosis was outside the scope of this study. In neural progenitor cells, activation of NMDARs causes transient recruitment of activator protein-1 (AP-1) to the DNA, followed with suppression in proliferation and induction of differentiation (29). In erythroid cells, AP-1 is known to be involved in regulation of proliferation and survival in Epo-dependent erythroid cells via controlling the activity of c-Jun and JunB transcription factors (23, 33).

Our results suggest that NMDARs may contribute to Epo-driven Ca^2+^ signaling along with TRP channels described earlier (15, 42, 44). Each of these channels mediating Ca^2+^ uptake respond to its own set of stimuli supporting a complex cross-talk between multiple receptors which drive the EPC differentiation process. Further studies are required to characterize the functional and possible physical interaction between these two types of channels.

Maturation to polychromatic and orthochromatic erythroblasts and finally to reticulocytes and RBCs is associated with a gradual replacement of this NMDAR type with the one mediating slowly decaying currents of smaller amplitude (Figs. 3 and 6, *B* and *C*). Induction of expression of the GluN2C subunit, which replaces GluN2A in receptors also containing the GluN2D and GluN3B subunits, coincides with the onset of hemoglobinization [*day 10* in culture according to Wickrema et al. (60) and Fig. 1]. Regulation of exocytosis of transferrin, its receptor recycling, and that of iron uptake by the EPCs in mice, rats, and rabbits is controlled by Ca^2+^/calmodulin and is bound to sense the changes in Ca^2+^ uptake mediated by the NMDARs (20). Apart from their possible involvement in iron handling at the later stages of EPC differentiation, “slow” NMDARs are best suited for the regulation of basal Ca^2+^ levels. Together with other Ca^2+^-permeable ion channels, such as voltage-dependent anion channels and voltage-gated calcium channel Ca_v_2.1, which have been characterized in RBCs (25), these channels contribute to the regulation of cell volume, redox balance control, proteolysis, and O_2_ affinity for hemoglobin (5, 18, 35).

#### Glutamate signaling in the microenvironment of EPCs.

Fluctuations of glutamate levels in peripheral blood are mirrored by alterations in the activity of erythroid NMDARs. Recently, we have shown that NMDARs in RBCs show a high degree of basal activity when in the circulation (18). Additionally, glutamate levels within the bone marrow may be regulated by controlled secretion of this amino acid from megakaryocytes and macrophages (3, 22, 32, 39). The close association of bone marrow and glutamatergic nerve endings may further contribute to the alterations in glutamate levels promoting erythropoiesis and increasing RBC production. However, the exact range of glutamate levels therein has never been measured. We have shown that hyperactivation of NMDARs in young and mature RBCs, particularly those in sickle cell disease patients, was associated with a transient increase in Ca^2+^, cell shrinkage, and oxidative burst (18). Prolonged chronic activation of NMDARs in EPCs causes receptor desensitization (Fig. 3*D*) but does not induce apoptosis in precursor cells (Fig. 8). We suggest that the desensitization is essential for cytoprotection as it controls Ca^2+^ uptake. Furthermore, Ca^2+^ oscillations mediated during the activation-deactivation cycles are used for signal transduction by a majority of cells (2, 51). The regulatory mechanisms and the physiological role of NMDARs expressed in EPCs as well as in other hematopoietic progenitors giving rise to leucocytes, and platelets require further investigation (28, 41). The pronounced toxic effect of NMDAR inhibition may be attributed to the importance of these receptors and Ca^2+^ fluxes they mediate for sustaining the EPCs' survival (42), but it more likely represents the off-target action of the receptor blockers at high concentrations (31).

In several studies, the receptors containing GluN2C/2D and GluN3B subunits were shown to have lower sensitivity to the channel pore blockers MK-801 and memantine and to Mg^2+^ block (7, 38, 54). Thus, the reduced efficiency of the pore-targeting MK-801 to inhibit NMDA currents is most likely associated with the unusual subunit compositions of erythroid NMDAR in which GluN1 is underrepresented, whereas GluN3B and 2D were most abundant. The low conductance and slow deactivation time we have observed for NMDARs in polychromatic erythroblasts were shown to be characteristic for the receptor formed by the GluN2C/D and GluN3B subunits (49). Currents mediated by the NMDAR in proerythroblasts showed no plateau phase as it was reported for the NMDAR currents in neuronal cells (4). These findings indicate that an atypical subunit composition and functional pattern of NMDAR in erythroid cells resemble the properties of the receptors described for other nonneuronal tissues (7, 28, 39).

#### Interindividual variability in quantity and quality of red blood cell production.

We have observed marked intercellular and interindividual variability in the expression pattern and abundance of NMDAR subunits in EPCs of healthy humans (Fig. 5, *B* and *C*). However, repetitive measurements performed for NMDARs in EPCs derived from CD34^+^ cells of the same donor were highly reproducible. Interindividual variability of the *GRIN* gene transcript levels could represent individual genetic, epigenetic, and signaling profiles (19). Intercellular heterogeneity in part reflects heterogeneity of the EPC maturation stages in culture. However, this heterogeneity in NMDAR abundance is persistent in circulating RBCs independent of cell age, suggesting that, despite similar morphology, several subpopulations of RBCs and EPCs are produced representing “glutamate-sensitive” and “glutamate-resistant” cells (18, 35). Expression patterns of *GRIN2A* and *GRIN2C* define the properties of the NMDARs as the levels in *GRIN2A* transcripts in the individual donors are proportional to the peak amplitudes of glutamate-sensitive currents and Ca^2+^ uptake, and are inversely proportional to the deactivation time duration (Figs. 5 and 6).

Based on the finding that enhanced Ca^2+^ influx is a trigger for increased terminal erythropoiesis (15, 42) we have suggested that GluN2A-containing NMDAR contributes to this essential Ca^2+^ uptake. This would imply that the interindividual variability in number of receptors per cell and its subunit composition might have an influence on red blood cell production, properties, and clearance (5). These observations concur with previous reports on the interindividual and inter-cellular variability in sensitivity to NMDAR agonists such as glutamate or homocysteine. The degree of glutamate-sensitivity of RBCs of healthy human individuals may contribute to the quality of stored blood products and the outcome of transfusion (35).

## GRANTS

The study was funded by a cooperative grant from ZIHP (Zurich Center for Integrative Human Physiology), University of Zurich (J. S. Goede, O. Speer, and A. Bogdanova), supported by the Vontobel Foundation (A. Bogdanova) and Hartmann-Müller Stiftung (J. S. Goede and O. Speer), and received funding from the European Community's Seventh Framework Programme (FP7/2007-2013) under grant agreement 602121 (CoMMiTMenT project) to A. Bogdanova.

## DISCLOSURES

No conflicts of interest, financial or otherwise, are declared by the author(s).

## AUTHOR CONTRIBUTIONS

P.H., P.J.K., M.S., O.S., and A.B. conception and design of research; P.H., V.T., J.S.G., and O.S. performed experiments; P.H., V.T., O.S., and A.B. analyzed data; P.H., V.T., P.J.K., O.S., and A.B. interpreted results of experiments; P.H. prepared figures; P.H., V.T., P.J.K., M.S., M.G., J.S.G., O.S., and A.B. edited and revised manuscript; P.H., V.T., P.J.K., M.S., M.G., J.S.G., O.S., and A.B. approved final version of manuscript; A.B. drafted manuscript.
